# The impact of involvement on researchers: a learning experience

**DOI:** 10.1186/s40900-017-0071-1

**Published:** 2017-09-18

**Authors:** Kristina Staley, Isabelle Abbey-Vital, Claire Nolan

**Affiliations:** 1 TwoCan Associates, Montague House, 4 St. Mary’s Street, Ross on Wye, HR9 5HT UK; 2 Parkinson’s UK, 215 Vauxhall Bridge Road, London, SW1V 1EJ UK

## Abstract

**Plain English summary:**

The impacts of involvement in research are often described in terms of the difference made to the research, the people involved and less frequently the researchers. This paper focuses on the researchers’ experiences of involvement, based on an evaluation of a pilot project supporting patient/carer involvement in research at Parkinson’s UK. Telephone interviews were conducted with researchers from eight different research projects with involvement. The researchers reported gaining new knowledge from patients and carers. They used this knowledge to change their project designs, interventions and new devices. They also gained new skills in communicating with the public. Meeting patients for the first time had a profound impact on some researchers, causing a change in their professional values. Face-to-face contact seemed particularly important to gain a sense of the ‘people behind the data’, which suggests such meetings may result in impacts beyond those typically achieved through an exchange of documents. Involvement also influenced one researcher’s choices and preferences, in terms of who to ask to take part in their study

In summary, researchers often learn something new from talking to patients and carers. Facilitating this conversation seems important to maximise the impact of this learning. In future, it might be helpful for evaluations of involvement to ask researchers in more detail about what they learnt from patients/carers and how they applied their new skills and knowledge. This may help to understand how involvement can influence researchers’ thinking to have an impact on research.

**Abstract:**

**Background**

The impacts of patient/public involvement are often described in terms of the difference made to the research, the researchers and the people involved. Involvement often impacts on *research* by influencing the design, delivery and dissemination. Patients/the public report gaining new skills and knowledge, increased self-confidence, and satisfaction from making a difference. There are fewer reports of the impacts on *researchers*. This paper discusses the findings from an evaluation of a pilot project supporting patient/carer involvement in research at Parkinson’s UK, focusing on the researchers’ experiences.

**Methods**

Semi-structured telephone interviews were conducted with one researcher from each of the eight research projects which involved patients/carers in the pilot. The findings were analysed using theoretical thematic analysis.

**Results and discussion**

Learning can be described as acquiring new knowledge, behaviours, skills, values, or preferences. The researchers’ reports reflected these different types of learning. They reported gaining new knowledge from patients and carers, which they recognised as distinct from their textbook knowledge of the condition. They used this learning to change their project designs and their new interventions and devices. They also gained new skills in communicating with patients and carers about the aims and significance of their research. Meeting patients for the first time had a profound impact on some researchers causing them to change their professional values. Face-to-face contact seemed particularly important to gain a sense of the ‘people behind the data’, which suggests such meetings may result in impacts beyond those typically achieved through an exchange of documents. The involvement also influenced one researchers’ priorities and preferences, in terms of what questions to ask and of whom, in their project.

**Conclusions**

Researchers learn from an exchange of knowledge with patients/ carers, which influences their plans and actions. This seems to be one way that involvement subsequently has an impact on research. Facilitating this exchange seems important to support mutual learning and to enhance the impact on researchers. Future evaluations of involvement might benefit from exploring what researchers learnt from patients/carers and how they applied their new skills and knowledge.

## Background

The impacts of patient/public involvement are often described in terms of the difference made to the research, the researchers and the involved patients/ members of the public [1–4]. Involvement often impacts on *research* by influencing the research question [5], the project design [6], the way the research is carried out [7], and the dissemination of the findings [8]. Patients/members of the public report gaining new skills and knowledge, increasing in self-confidence, and gaining satisfaction from making a difference [9]. By way of contrast, there are far fewer reports of the impacts on *researchers*. The most commonly reported impact is a requirement for more resources and for more of the researchers’ time, sometimes slowing the pace of research.

The contributions made by patients/ the public during their involvement are informed by their experiential knowledge. i.e. what they have learnt from living with a health condition and/or using services. Patients/the public’s knowledge, insights and perspectives will be ‘new’ to researchers [10]. Researchers will often therefore *learn* from their experience of involvement. What researchers learn changes their practice, which may be one mechanism by which involvement leads to the reported outcomes for research [11].

This article reports on an evaluation of a pilot project of patient and carer involvement in research conducted by Parkinson’s UK. The evaluation aimed to gather learning as to what had worked well and what might still be improved, as well as what difference the involvement had made. The staff at Parkinson’s UK offered training to the volunteers and support to the researchers prior to an initial meeting, where the researchers received feedback on their research proposals from patients and carers. A number of the researchers continued to work with patients/carers to finalise a funding application or develop their therapeutic intervention. Therefore all stakeholders (researchers, patients, carers and staff) were asked for their views on the process and impact of the involvement. The findings from the pilot are reported elsewhere [12]. Parkinson’s UK used the findings to further develop their PPI practice and policy.

In this article, we discuss what the evaluation revealed about researchers’ experience of involvement and in particular what they learn from the process. Learning can be described as ‘the act of acquiring new knowledge, behaviours, skills, values, or preferences’ [13]. We therefore reflect on how the involvement contributed to the researchers gaining new knowledge and skills, and how this shaped the researchers’ behaviour, values and preferences in relation to their research.

## Methods

For the purpose of the evaluation, semi-structured telephone interviews lasting 45–60 min, were conducted with one researcher and one patient or carer from each of the eight research projects that took part in the pilot. Seven of the eight projects involved clinical research and one was basic science. The researchers included both junior and senior members of the respective research teams from England, Scotland and Wales. Some were looking for input from patients/carers to inform the development of a funding bid, while others were looking for help with developing a new device or therapeutic intervention. In this article, we focus on the researchers’ reports of their experience of the involvement, drawing on the interviews with the researchers. The patients/carers were asked about their own experiences of being involved in the pilot, which were somewhat different to the researchers’, and these findings are included in the full evaluation report [12].

The evaluation was supported by Parkinson’s UK’s Involvement Steering Group which includes people affected by Parkinson’s and Parkinson’s researchers. The Group advised on the selection of interviewees and helped shape the interview questions. One area the Steering Group were keen to explore was the researchers’ experience of involvement, in particular the researchers’ feelings and thoughts before and after involvement, and the personal impacts. Other questions asked about the reasons for involving patients/carers, the researchers’ expectations of involvement, their views on the support from Parkinson’s UK and the impacts on the research.

The interviews were carried out by KS between February and March 2016. With the interviewee’s permission, the interviews were recorded and transcribed. The transcripts were initially analysed using inductive thematic analysis [14], (i.e. the themes were shaped by the interviewees’ responses) to identify the impacts on the research and to draw out lessons for Parkinson’s UK. KS carried out the initial thematic analysis which was checked and confirmed in meetings with IAV and CN and members of the Steering Group. These findings are discussed in the evaluation report [12].

We carried out a second analysis of the researchers’ interview transcripts, using theoretical thematic analysis i.e. based on a theory that researchers are learning from involvement [11]. We therefore looked for statements in the interviews where researchers reported gaining new knowledge or skills, and/or a change in values, preferences and behaviour. KS carried out the initial thematic analysis which was checked and confirmed by IAV and CN. The different aspects of the researchers’ learning are discussed in the combined results and discussion section.

## Results and discussion

### Patients and carers provide new knowledge and understanding

Many of the researchers reported that working with patients and carers had given them a better understanding of life with Parkinson’s and the issues that matter to the people who are affected. They recognised that patients’ and carers’ experiential knowledge was different to their text-book knowledge of the condition.

*“I see them as being experts in experiencing either Parkinson’s or caring for someone with Parkinson’s and so… they can add things and views that perhaps I wouldn’t, or the research team may not have considered… rather than us [the researchers] assuming…we’re working as a team collaboratively with these people, they’ve got something else to bring to it that you can’t”.* Researcher 1

*“It was easy for me to read up on the mechanisms behind Parkinson’s, so the degeneration and how it happens in the brain. That’s the literature… what I learnt from working with patients and carers are the more day to day aspects or difficulties that come with Parkinson’s disease. I don’t know anyone with Parkinson’s, so that was new to me”.* Researcher 2

The majority of the researchers in this pilot used this new understanding of Parkinson’s to improve the practical design of their projects and make it easier for patients to participate i.e. this knowledge changed their choices around how they planned to conduct their research. For example, one researcher had not anticipated that patients would have any difficulty taking part in telephone interviews, until the patients explained that people with advanced Parkinson’s may have a weak voice. The researcher therefore introduced a range of mechanisms by which patients could contribute their views.

Similarly, an engineer seeking input into the design of a feasibility trial of a new device to improve posture, learnt how the symptoms of Parkinson’s might prevent patients from being able to use the device, and how it would need to be changed to make this possible. For example, double-sided tape was used to stick the device to the person’s back, which some patients were unable to manage. These changes were small, but extremely important to make the device practical and useful.

*“It was a bit of an eye-opener for me, and it brought me more in line with what [patients] would deal with on a daily basis... It was really small things that I would have taken for granted… things that you would almost ignore, really, in the design of the trial.* Researcher 7

In the lead up to the meetings with the Parkinson’s UK volunteers, the researchers had inevitably developed their ideas in the absence of patients’/carers’ knowledge. They had therefore naturally made assumptions about what would be important or acceptable to patients/ carers. The involvement seemed to have a particularly significant impact when these assumptions were challenged, and researchers learnt them to be incorrect. This insight gained through involvement is often described as a ‘lightbulb moment’ [15, 16]. For example, in one of the projects, the researcher required participants to complete a large number of questionnaires, some of which asked potentially distressing questions. The researcher anticipated that this would be demanding of patients and assumed it might be important to seek input on the design and format of the questions and to ask who might best administer the questionnaires. While the patients/ carers did make these kinds of comments, they also explained that they would need to understand the significance and value of the questions being asked, in order to feel motivated to complete the task. This was a point of mutual learning.

*“None of the people [patients and carers] round the table really had a concept of what the study wanted to do… so there was a failure somewhere in our communicating that message… so when asking someone to complete a questionnaire, they would be wondering ‘What’s this all about?’... So each time we need to explain to them why the questionnaire is here and what are we going to do with the information once we get it, that’s what I learned in the meeting… At one point I said ‘We study every single piece of information that we’re collecting…there’s nothing redundant in here’. One patient said ‘That’s astonishing but that’s not clear. It’s just page after page of questions, so you just think a lot of this will go to waste.’ That was a penny drop moment for me…”* Researcher 5

Based on previous experience of evaluating involvement, the researchers who have experienced such a ‘lightbulb’ moment tend to have a deeper understanding of the value of involvement. They are often the people who state they would not now consider conducting any of their research without involving patients/ carers [17].

### Researchers gain new skills from involvement

In preparation for the meetings with patients and carers, Parkinson’s UK staff supported the researchers in preparing their presentations and summaries of their research to send out in advance. During the meetings, the patients and carers often provided input into the researchers’ lay summaries as well as other written materials (posters, questionnaires and patient information sheets). Patients/carers not only commented on the language used but also the content. It seemed that in initially drafting the written information, researchers had made assumptions about what potential participants would want to hear. The involvement revealed what patients and carers would actually want to know. In this way, the involvement led to an improvement in the researchers’ communication skills and their ability to explain their research to an audience of patients and carers.

*“There was good stuff around wording of the patient information sheet… we hadn’t mentioned what most people with Parkinson’s disease focus on… we’d skipped over the obvious thing and gone straight into detail and that didn’t make sense to people…”* Researcher 4

### Changes in researchers’ values

Some researchers expressed surprise at learning that patients/carers were so enthusiastic about research. This was particularly true for the junior researchers, who met patients and carers for the first time. This experience had a profound impact. It caused one researcher to re-evaluate his career, and another to conclude that people in his profession should change their approach to their work. The involvement thus caused a shift in their personal and professional values.

*“There is a personal aspect that must not be understated. You realise that the work that you’re doing is very important to people… sometimes when you work, things don’t go your way, there’s problems. It gets really frustrating and you think ‘What’s the point?’ The pay isn’t great, the funding is hard to come by, the job security is sometimes difficult… but then I realise… there is actually quite a lot of importance behind the stuff that I do for people – that is of value to me – it makes me feel good about what I’m doing”.* Researcher 2

*“If I design and I make something as an engineer, then I would think there’s nothing wrong with this - I built it, so obviously it’s absolutely perfect! But things don’t work like that. I’m much more aware now that you can never account for all the intricacies that might actually happen on a daily basis when people go to actually use such a thing. So there would be value in taking people who are from a very technical engineering background and broadening their horizons to deal with people [patients and carers], to learn about their likes and dislikes, and find out what they [patients and carers] think pragmatically about any kind of technology or any kind of device that you’re trying to bring to the clinic”.* Researcher 7

### Changes in researchers’ preferences or priorities

All of the researchers who took part in the pilot came with clear ideas of their research topic and their research question, because they had already drafted detailed proposals or were halfway through their study. Therefore there was limited scope for involvement to influence researchers’ choices and preferences i.e. the thinking behind a study. However, in one case, the involvement did seem to influence the researchers’ preferences or priorities, resulting in changes to the conceptual design. The researcher had originally planned to interview only patients with Parkinson’s about their memory and cognitive problems. The patients and carers he spoke to pointed out that carers might have a different perspective on how much their loved-one is affected, and could make an important contribution to the study. As a result, the researcher adapted his methodology to include interviews with carers as well as patients.

## Conclusion

The findings from this evaluation of a pilot study at Parkinson’s UK appear to support the conclusions drawn from a previous narrative review of the involvement literature [11], namely that researchers acquire new skills and knowledge through involvement, which leads to changes in their values, preferences and practice. It is these changes that may underpin many of the impacts of involvement on research when involvement takes the form of collaboration between researchers and patients/carers.

Shifting the focus of the impact debate from describing the impacts on research to the impacts on researchers and what they learn from involvement, may be important to facilitate a deeper understanding of how involvement works and therefore ideas about how to improve practice. Learning requires an exchange of knowledge, which places greater emphasis on supporting a constructive dialogue between researchers and patients/ carers and facilitating their interaction. It is of note that the researchers and patients/carers involved in this pilot study placed great value on meeting face-to-face and being able to answer each other’s questions [12]. The facilitation from Parkinson’s UK staff proved essential to ensuring a positive and focused exchange. The researchers’ also reported that meeting patients and carers in person was vital to their realisation of there being ‘people behind the data’, and seemed to contribute to some of their reports of a profound shift in their attitudes to their work.

By way of contrast, a focus on the impacts on the *research* tends to place greater emphasis on the objective outputs, rather than the researchers’ personal experience. With this understanding, it becomes possible to reduce involvement to an exchange of documents, asking a remote panel of patients and carers to comment on patient information sheets and protocols without any interaction with researchers. While this process can be very effective and provide useful input, based on the findings presented here, it may limit the extent of the impact of involvement and the opportunities for valuable mutual learning.

However, we would not want to conclude that all involvement requires face-to-face meetings, more that it may be important to prepare and support researchers and patients/carers to be able to exchange knowledge and ideas. This may help to challenge assumptions and cause a shift in attitudes in a way that an email exchange of documents might not. As not all patients/ carers are able to travel, it might also be important to use other mechanisms, e.g. teleconferences and skype, or even home visits by researchers, to enable such a dialogue to take place. In addition, it seems that the support of a facilitator is key in preparing researchers to listen, preparing patients/carers to share the most relevant aspects of their experiential knowledge and to challenge constructively, and enabling the conversation to take place when the two parties may not speak the same language.

### Implications for future evaluations of involvement

This evaluation was shaped by Parkinson’s UK’s Involvement Steering Group and the evaluator’s experience of conducting similar evaluations [17, 18]. The interview schedules for researchers, patients/carers and staff included standard questions about expectations, process, impacts on the research and on the individuals involved, challenges and barriers, and finally ideas for improvements. However, no questions were asked explicitly about learning. Based on the findings, it may be valuable in future evaluation projects to ask researchers in particular about what they learnt from involvement and how they applied their learning. Such an approach may provide greater insight into how the involvement led to the impacts on research, and encourage the individuals involved to reflect on their experience to develop their involvement practice.

### Limitations of this study

The findings from this study are based on only a small number of interviews with mainly clinical researchers working on only one health condition. It would be important to conduct further research to explore whether the findings are generalisable across other fields and areas of research.

We note that there is a vast literature on ‘learning’ that we have not explored as we are not experts in the theory of learning. The early ideas presented here would benefit from being further conceptualised to better define what learning means in the context of involvement in research. We hope this work will stimulate further research in this area.

## Abbreviations

UK: United Kingdom
